# Supplementary material for: Serine auxotrophy is a targetable vulnerability driven by PSAT1 suppression in AML
Source: bioRxiv. 2025 May 14:2025.05.13.651470. Preprint. [Version 1] doi: 10.1101/2025.05.13.651470 (PMC12132333; doi:10.1101/2025.05.13.651470)
Supplement: Supplement 1 [file media-1.pdf]

Supplementary Materials

Supplementary Methods p2-5

Supplementary Tables p6-8

Supplementary Figures p9-15

References p16

Supplementary Methods

Cell lines and AML patient samples: THP1, KASUMI3, KASUMI4, KASUMI6, AML193, TF1, KG1, U937, HEL9217, GDM1, and OP9 were purchased from ATCC. KASUMI1, HL60, K562, MOLM13, MONOMAC6, and MV411 were gifts from LP Gondek (Johns Hopkins University). SKM1, F36P, OCIAML3, OCIAML5, PL21, OCIAML4, MUTZ3, HNT34, and UCSDAML1 were purchased from DSMZ. KO52 was purchased from AcceGen. P31FUJ was purchased from the JCRB cell bank. OCIAML20 was a gift from M Minden (Princess Margaret Cancer Centre)(1). YCUAML1 was a gift from H Kunimoto (Yokohama City University) (2). STR authentication and mycoplasma testing were performed for all cell lines. Primary samples were collected from the bone marrow or blood of AML patients who provided written consent under the J0969 institutional review board–approved cell bank protocol at Johns Hopkins University. In all primary cultures, high blast number (>75%) was confirmed by a CD45-dim, low-side scatter phenotype by flow cytometry.

Cell line transcriptomic analysis. For auxotrophic and non-auxotrophic (as defined in Figure 1) cell lines that were also part of the Cancer Cell Line Encyclopedia, mRNA gene expression profiles of the two groups were subjected to a custom two-class comparison in the DepMap portal (https://depmap.org/portal/). A volcano plot of the difference in gene expression (auxotrophic log2(TPM1+1) – non-auxotrophic log2(TPM1+1)) versus -log10(p-value) was created using PRISM.

Serine starvation studies: For -S studies, we used two media: commercially available serine-, glycine-, and dextrose-free RPMI from Teknova, and customized serine- and glycine-free HPLM from ThermoFisher. For RPMI, 11 mM dextrose, 133 µM glycine, +/- 286 µM L-serine were added back to the media, along with 20% dialyzed FBS (dFBS) and 1% Pen/Strep. For HPLM, 300 µM glycine +/- 150µM L-serine were added back, along with 5% dFBS and 1% Pen/Strep. Growth factors were then added as in master cultures per above. Cells were seeded at 1×10^5 cells/mL for cell lines and $2–3 \times 10^5$ cells/mL for primary AML samples in flat-bottom 96-well plates, in triplicate or quadruplicate. To prevent cell death under -S conditions, 1×10^6 HNT34 cells were seeded into 6-well plates and incubated for 3 days in -S or +S media, with or without 25 µM pan-caspase inhibitor QVD. On day 3, the cells were resuspended in

Propidium Iodine (3 μg/ml) and run on an LSR-II flow cytometer. Exponential (Malthusian) growth constants (K) were calculated using PRISM (GraphPad).

Western blotting: Total protein was extracted from 1 × 10^6 cells in RIPA lysis buffer supplemented with a protease/phosphatase inhibitor cocktail (HaltProtease and Phosphatase Inhibitor Cocktail). Protein concentration was determined using the bicinchoninic acid (BCA) protein assay per the manufacturer's protocol (ThermoFisher Rapid Gold BCA Protein Assay Kit). Equal amounts of protein (30μg) were resolved by Novex NuPAGE SDS-PAGE and transferred to low-fluorescent polyvinylidene fluoride (PVDF) membranes using a semi-dry transfer method (Invitrogen PowerBlotter). The membranes were then probed with antibodies against PSAT1 (1:1,000), PHGDH (1:1,000), ATF4 (1:500), GAPDH (1:2,000), COX-IV (1:5,000), and MGST1(1:500). A secondary goat anti-rabbit (1:15,000) was used. Immunoreactive bands were visualized using ECL or ECL+ reagent. WB densitometry was performed as describe in the literature (3).

DNA constructs: PSAT1 lentiviral constructs (Neo-EF1A-hPSAT1-T2A-TurboGFP) were designed to express Neomycin resistance, TurboGFP, and either the wild-type (WT) or enzyme-dead, structurally intact K200A (mut) PSAT1 protein. For PHGDH, constructs (Bsd-EF1A-hPHGDH-IRES-EBFP) expressed Blasticidin resistance, EBFP, and either WT or enzyme-dead D175N/R236K/H283A (mut) PHGDH protein. Lentiviral construct for overexpression of MGST1 was synthesized by VectorBuilder to express Neomycin resistance and TurboGFP along with human MGST, and the plasmid structure was the following: Neo-EF1A-hMGST1-IRES-TurboGFP. Constructs were synthesized by VectorBuilder. For PSAT1 knockouts, 3 Lentiviral constructs were synthesized by VectorBuilder carrying scrambled and two gene-targeted guide RNAs (gRNA30 and gRNA_exon1)(4) in the following structure: U6-gRNA-EFS-hCAS9-T2A-Puro.

Mass spectrometry quantification of serine synthesis: For HNT34 double rescued PSAT1 and PHGDH cells, 3 x 10^6 cells were seeded in flasks and cultured under normal conditions for 2 days. On day 4, the media was replaced with fresh media supplemented with ^{13}C-glucose. Cells were collected and centrifuged at 500 g for 5 mins at 4 degrees Celsius. The media was carefully removed, and the cell pellets were washed three times with 1 mL of PBS before being transferred to 1.5 mL safe-lock Eppendorf tubes. The cell pellets were centrifuged again at 500 g for 5 mins at

4 °C and the PBS supernatant was gently removed. Tubes then were snap-frozen in liquid nitrogen for 1 min and stored at -80°C. For controls, unlabeled cells for each cell clone, as well as parental HNT34 cells, were included. The same sample processing was performed for auxotrophic and non-auxotrophic cell lines (MUTZ3, YCUAML1 P31FUJ, UCSDAML1, MOLM13, OCIAML3, OCIAML5). Polar metabolites were extracted from samples with 80% methanol as the extraction solution. Polar metabolite-containing supernatant was isolated after centrifugation and dried under nitrogen gas. Dried metabolite extracts were resuspended in 50% methanol and then clarified by centrifugation at 15,000 g and 4 °C for 15 min. Finally, the supernatant was transferred into the 250ul sample vial and injected into the liquid mass spectrometry (LC-MS) system for analysis. LC–MS based metabolomics profiling was performed on a Vanquish UHPLC system (Thermo Fisher, Waltham, MA, USA) coupled with an Orbitrap Q Exactive™ HF-X mass spectrometer (Thermo Fisher, Waltham, MA, USA) at Complete Omics (Baltimore, MD, USA). The samples were injected into a Waters XBridge BEH Amide column (2.1 x 150 mm, 1.7μm) using a 25-min gradient at a flow rate of 0.2 mL/min. The LC parameters were as follows: autosampler temperature, 4 °C; injection volume, 2 ml; column temperature, 40 °C; and flow rate, 0.20 ml/min. The solvents and optimized gradient conditions for LC were: Solvent A, Water with 0.1% formic acid; Solvent B, Acetonitrile with 0.1% formic acid; A non-linear gradient from 99% B to 45% B in 25 minutes with 5min of post-run time. The QE HF-X mass spectrometer was operated in the negative mode with the following optimized operation parameters: Sheath gas flow rate, 35 arb; Aux gas flow rate, 10 arb; Sweep gas flow rate, 2 arb; Spray voltage, 2.85 kV; Capillary temperature, 350 °C. Data were acquired with Xcalibur acquisition software and processed with Compound Discoverer 3.3 (CD ver. 3.3, Thermo Fisher). The analyte database used for metabolites annotation was developed in-house with retention times based on the LC method described as above. The stable isotope labeling data were processed by the 'StableIsotopeLabeling' workflow unit of the Compound Discoverer software.

***In vivo*:** Animal experiments were approved by the Johns Hopkins University Animal Care and Use Committee. Six- to eight- week-old female NOD-SCID IL2Rgnull (NSG) mice were used for all *in vivo* experiments except of the experiment depicted in Figure 5C in which 6-week-old female NOD-scid IL2Rgnull-3/GM/SF (NSGS) mice were used. For leukemia burden assessment, peripheral blood (PB) was collected via tail vein bleeding on day 14, and then mice were euthanized for Bone Marrow (BM) and spleen harvest. BM cells were isolated from the femurs. The spleen, PB, and BM cells were incubated in the lysing buffer for 15 min × 3, then passed through a strainer into flow cytometry

tubes. Cells were stained using anti-mouse CD45 conjugated with PE/Cy7 and anti-human CD45 conjugated with APC. Live/dead staining was performed using PO-PRO-1. Data analysis was performed using FlowJo software by directly comparing single human CD45$^+$ and single mouse CD45$^+$ events. Venetoclax was given orally in a total volume of 200 µL using a 20G feeding tube. The Venetoclax formulation consisted of 10% DMSO, 30% PEG400, and 60% Phosal-PG50.

Methylation-specific qPCR: Genomic DNA was isolated from 1 million cells using a genomic DNA extraction kit from New England Biolabs and subjected to bisulfite conversion using the EZ DNA Methylation-Gold Kit from Zymo Research. Primer pairs for unmethylated (unmeth) and methylated (meth) DNA were designed and validated for the correct size and methylation status through gel electrophoresis and Sanger sequencing. The qPCR was run in triplicate and captured on a BIORAD CPX96. The protocol is as follows: Initial denaturation: 95°C for 3 minutes, amplification: Denaturation: 95°C for 10 sec, annealing: 58.9°C for 30 sec (primers bind to target sequences). The total number of cycles was 45. Melt Curve Analysis: Increase by 0.5°C every 5 seconds starting from 65°C to 95°C

Transcriptome and methylation analysis of primary AML cohorts. The expression of PSAT1 mRNA levels according to different genetic aberrations in AML was performed by comparing RPKM levels obtained from cBioportal (https://www.cbioportal.org) for the TCGA and BEAT-AML cohorts. For the Leucegene cohort, TPM values and genetics were obtained from MISTIC (http://mistic.iric.ca), the Leucegene site (https://leucegene.ca), and GEO series GSE67040. Significance was assessed with unpaired t-tests on Box-Cox-transformed RPKM or TPM levels from the two groups (lambda optimized for normality by Kolmogorov-Smirnov testing). PSAT1 methylation values for comparison with PSAT1 mRNA were obtained from cBioportal for TCGA and from Giacopelli et al for BEAT-AML (5).

Supplementary Table 1

PSAT1 KO guides	
sgRNA30 (KO_1)	CTTTCACAGAAATGAGTCAC
sgRNA_exon1 (KO_2)	CTTACTGAGTGCGGCAGCT (4)
**PSAT1 methylation specific primers**	
Methylated PSAT1 Forward primer	5′- GTAGGGTTTGCGATAGTACGG -3′ (ref. 6)
Methylated PSAT1 Reverse primer	5′- GCTACGATAAAAATCTACAACCGAC -3′ (ref. 6)
Unmethylated PSAT1 Forward primer	5′- TAATTAGTGTAGGGTTTGTGATAGTATG -3′
Unmethylated PSAT1 Reverse primer	5′- AAAAATCTACAACCAACCCCAA -3′

Supplementary Table 2

Item	Brand	Catalog Number
Culturing		
Fetal Bovine Serum (FBS), Premium, heat-inactivated	Thermo Fisher Scientific	A5670801
Fetal Bovine Serum, dialyzed, US origin	Thermo Fisher Scientific	26400044
Serine	Millipore Sigma	S4311
Glycine	Millipore Sigma	G8790
Dextrose	Millipore Sigma	D9434
Penicillin – Streptomycin	Thermo Fisher Scientific	15140122
RPMI 1640 Medium	Thermo Fisher Scientific	11875093
RPMI-1640 Media without Glucose, Glycine and Serine. 500mL, Sterile	Teknova	R9660-02
Human plasma-like medium (HPLM)	Thermo Fisher Scientific	ME22541L1
Human IL-6 Recombinant Protein, PeproTech®	Prepro tech / thermo	200-06
Human IL-3 Recombinant Protein, PeproTech®	Thermo Fisher Scientific	200-03
Human TPO (Thrombopoietin) Recombinant Protein, PeproTech®	Thermo Fisher Scientific	300-18
Human SCF, Animal-Free Recombinant Protein, PeproTech®	Thermo Fisher Scientific	AF-300-07
Human Flt-3 Ligand (FLT3L) Recombinant Protein, PeproTech®	Thermo Fisher Scientific	300-19
Recombinant Human GM-CSF	Thermo Fisher Scientific	300-03
UM729	Stemcell Technologies	72332
StemRegenin 1 (Hydrochloride)	Stemcell Technologies	72352
Vi-CELL BLU Reagent Kit	Beckman Coulter	C06019
Genetics		
SYBR™ Green Universal Master Mix	Thermo Fisher Scientific	4309155
EZ DNA Methylation-Gold Kit	Zymo Research	D5005
Monarch® Genomic DNA Purification Kit	New England Biolabs (NEB)	T3010L
Animal experiments		
HBSS	Thermo Fisher Scientific	14025092
Baker Amino Acid 1/2" Pellet IRR (5CC7) 5kg	Animal Specialties And Provisions	1812426
Mod TestDiet 5CC7 w/ No added Serine or Glycine 1/2" Pellet IRR (5BJX) 5kg	Animal Specialties And Provisions	1817070-203
Venetoclax	Selleck Chemicals	S8048
Dimethyl sulfoxide	Millipore Sigma	D2650
PEG400	Selleck Chemicals	S6705
Phosal 50 PG	MedChem Express	HY-Y1903
ACK Lysing Buffer	Quality Biological	118-156-101
Flow cytometry staining		
PE/Cy7 anti-mouse CD45 [30-F11]	BioLegend	103114
APC Mouse Anti-Human CD45	BD Bioscience	555485
PO-PRO™-1 Iodide (435/455), 1 mM Solution in DMSO	Thermo Fisher Scientific	P3581
eBioscience™ Propidium Iodide	Thermo Fisher Scientific	BMS500PI
Western Blotting		
Psat1 Polyclonal Antibody	Thermo Fisher Scientific	PA5-22124
Anti-PHGDH Antibody	Atlas Antibodies	HPA021241
ATF-4 (D4B8) Rabbit mAb	Cell Signaling Technology	11815S
GAPDH (D16H11) XP® Rabbit mAb	Cell Signaling Technology	5174S
MGST1 Recombinant Rabbit Monoclonal Antibody (6A0S5)	Thermo Fisher Scientific	MA5-42631
COX IV (3E11) Rabbit mAb	Cell Signaling Technology	4850
RIPA Lysis and Extraction Buffer	Thermo Fisher Scientific	89900

Halt™ Protease and Phosphatase Inhibitor Cocktails, Thermo Scientific, Halt Protease and Phosphatase Inhibitor Cocktail, (100 X)	Thermo Fisher Scientific	78440
Pierce™ Rapid Gold BCA Protein Assay Kit	Thermo Fisher Scientific	1863381
NuPAGE™ LDS Sample Buffer (4X)	Thermo Fisher Scientific	NP0007
NuPAGE™ Sample Reducing Agent (10X)	Thermo Fisher Scientific	NP0009
NuPAGE™ Bis-Tris Mini Protein Gels, 4–12%, 1.0–1.5 mm	Thermo Fisher Scientific	NP0336BOX
NuPAGE™ Bis-Tris Mini Protein Gels, 4–12%, 1.0–1.5 mm	Thermo Fisher Scientific	NP0321BOX
NuPAGE™ Bis-Tris Midi Protein Gels, 4 to 12%, 1.0 mm	Thermo Fisher Scientific	WG1402BOX
Power Blotter Select Transfer Stacks, PVDF, regular size	Thermo Fisher Scientific	PB5310
Power Blotter 1-Step™ Transfer Buffer (5X)	Thermo Fisher Scientific	PB7100
Invitrogen™ NuPAGE™ MOPS SDS Running Buffer (20X)	Fisher Scientific	NP0001
SuperBlock™ Blocking Buffer	Thermo Fisher Scientific	37536
Signalfire™ ECL Reagent	Cell Signaling Technology	6883P3
Signalfire™ Plus ECL Reagent	Cell Signaling Technology	12630S
Pierce CL-Xposure™ Film, Thermo Scientific, CL-XPosure™ Film, Packaging=100 Sheets, Dimensions=12.7 x 17.8 cm (5 x 7)	Thermo Fisher Scientific	34090
Anti-rabbit IgG (H+L) (DyLight 680 Conjugate)	Cell Signaling Technology	5366P
Cell transduction and Selection		
LentiBOOST® Research Grade Lentivirus Transduction Enhancer Solution	Mayflower Bioscience	SB-P-LV-101-03
Puromycin Dihydrochloride	Thermo Fisher Scientific	A1113803
Geneticin Selective Antibiotic (G418 Sulfate)	Thermo Fisher Scientific	10131035
Corning® 50 mg Blasticidin S HCl	Corning	30-100-RB
Software		
GraphPad	Dotmatics	https://www.graphpad.com
FlowJo	BD	https://www.flowjo.com/
SnapGene	Dotmatics	https://www.snapgene.com
MethPrimer	Ractigen Therapeutics	https://www.methprimer.com
ApE	M. Wayne Davis/ University of Utah	https://jorgensen.biology.utah.edu/wayned/ape/
Fiji	Johannes Schindelin and collaborators	https://fiji.sc
Compound Discoverer™	Thermo Fisher Scientific	https://www.thermofisher.com/us/en/home/industrial/mass-spectrometry/liquid-chromatography-mass-spectrometry-lc-ms/lc-ms-software/multi-omics-data-analysis/compound-discoverer-software.html
Microsoft Excel	Microsoft	https://www.microsoft.com/en-us/microsoft-365/excel/
R	R Core Team	https://www.r-project.org

Supplementary Figure 1

A

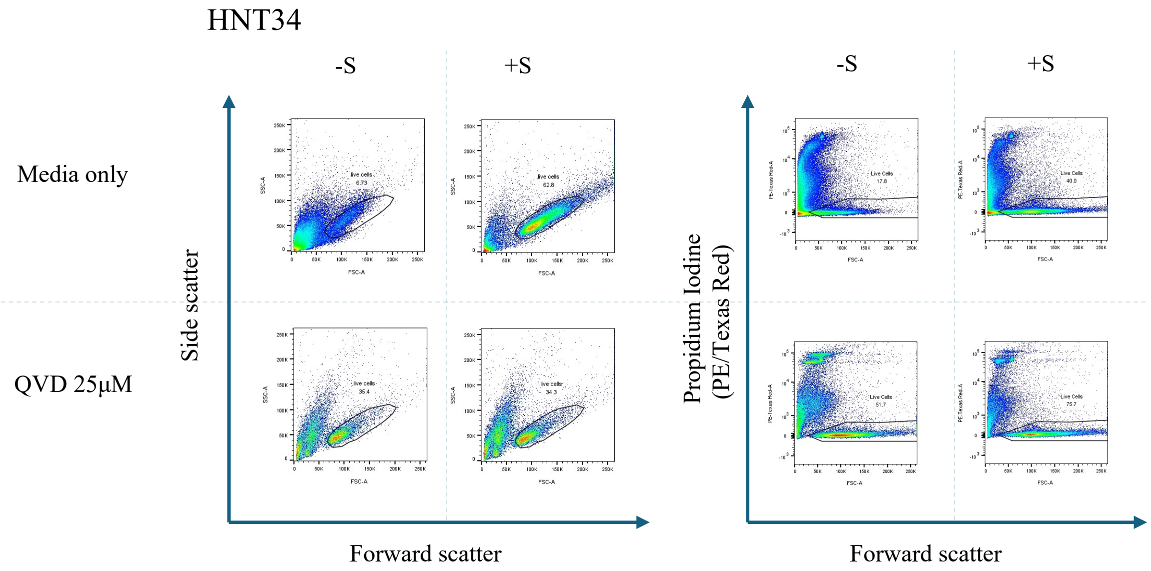

B

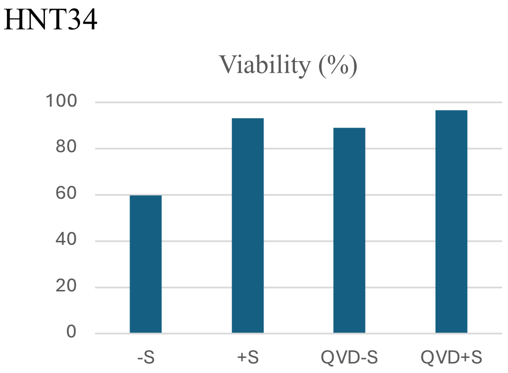

Figure S1. (A) Flow cytometry analysis of HNT34 cells after 3-day incubation in +S or –S RPMI, in the presence or absence of QVD (25 µM). The final DMSO concentration in QVD-treated conditions was 2.5%. Live cells were gated based on forward and side scatter or forward scatter and Propidium Iodine (PE/Texas Red channel) (B) Viability of HNT34 in the same conditions, assessed by trypan blue exclusion using the Vi-CELL BLU.

Supplementary Figure 2

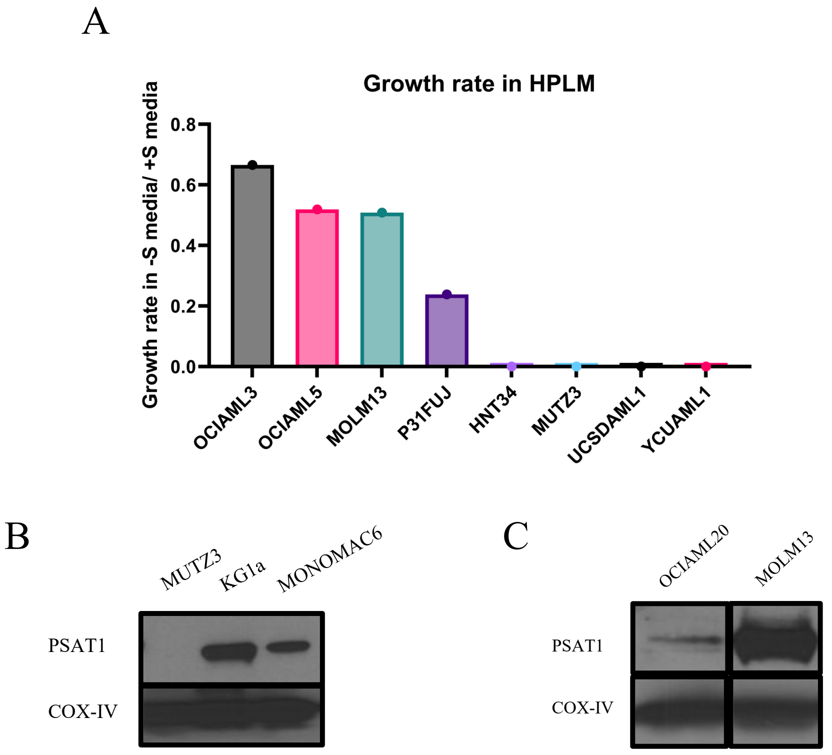

Figure S2. (A) AML growth rate (Malthusian constant; K) ratio as measured in starvation studies in -S and +S HPLM. Cell lines were run in triplicate or quadruplicate in +S and –S conditions for growth rate calculation. (B) Validated PSAT1 expression levels of MONOMAC6, using COX-IV as a loading control. KG1a serves as a positive and MUTZ3 as a negative PSAT1 control. (C) Increased sensitivity western blot showing limited PSAT1 expression in OCIAML20. MOLM13 was used as a PSAT1 positive control.

Supplementary Figure 3

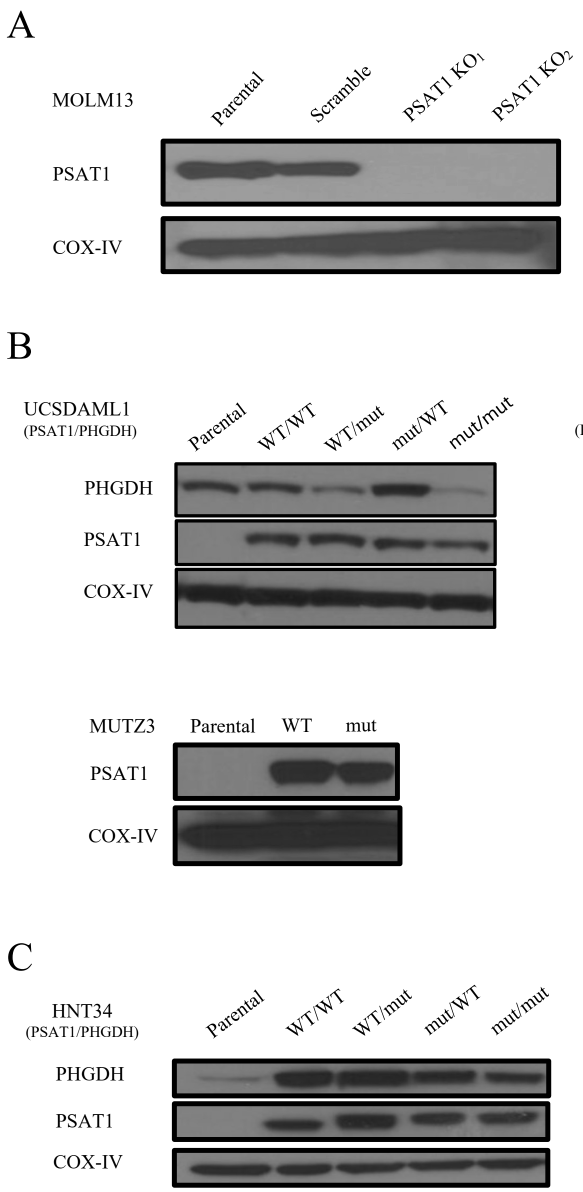

Figure S3. Western blot validation of (A) protein downregulation for PSAT1 in MOLM13 PSAT1 Knockout, and (B-C) upregulation of PSAT1 (mut or WT) in UCSDAML1, P31FUJ, MUTZ3, PL21, and HNT34, and PHGDH (mut or WT) in UCSDAML1, P31FUJ, and HNT34 transduced models. (B-C) “Mut” refers to inactivation mutations of the PSAT1 or PHGDH protein.

Supplementary Figure 4

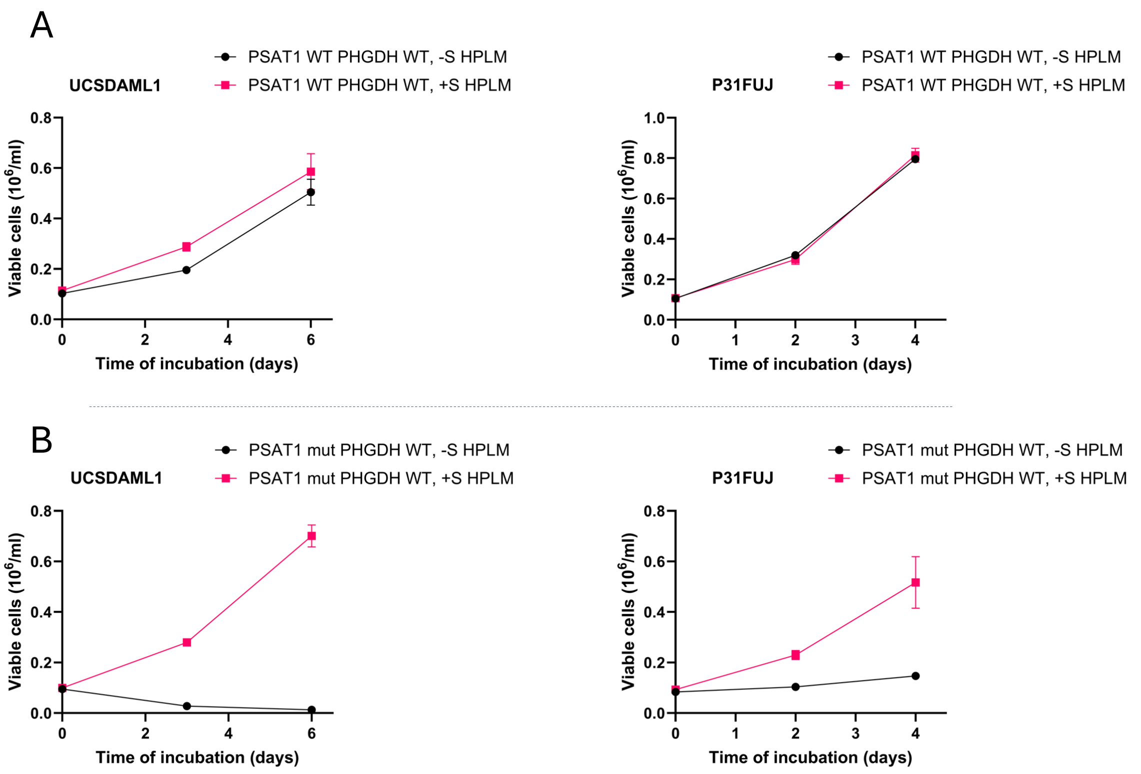

Figure S4. PHGDH overexpression has no additive growth advantage in cells in serine-depleted conditions. (A) Compared to the PSAT1 WT or mut rescue depicted in main Figure 4B, overexpression of PHGDH in UCSDAML1 and P31FUJ cells shows no additional growth benefit in –S HPLM. (B) PHGDH overexpression alone in UCSDAML1 and P31FUJ cells does not enhance growth in –S HPLM. Each sample was run in quadruplicate. "Mut" refers to inactivation mutations of the PSAT1 protein.

Supplementary Figure 5

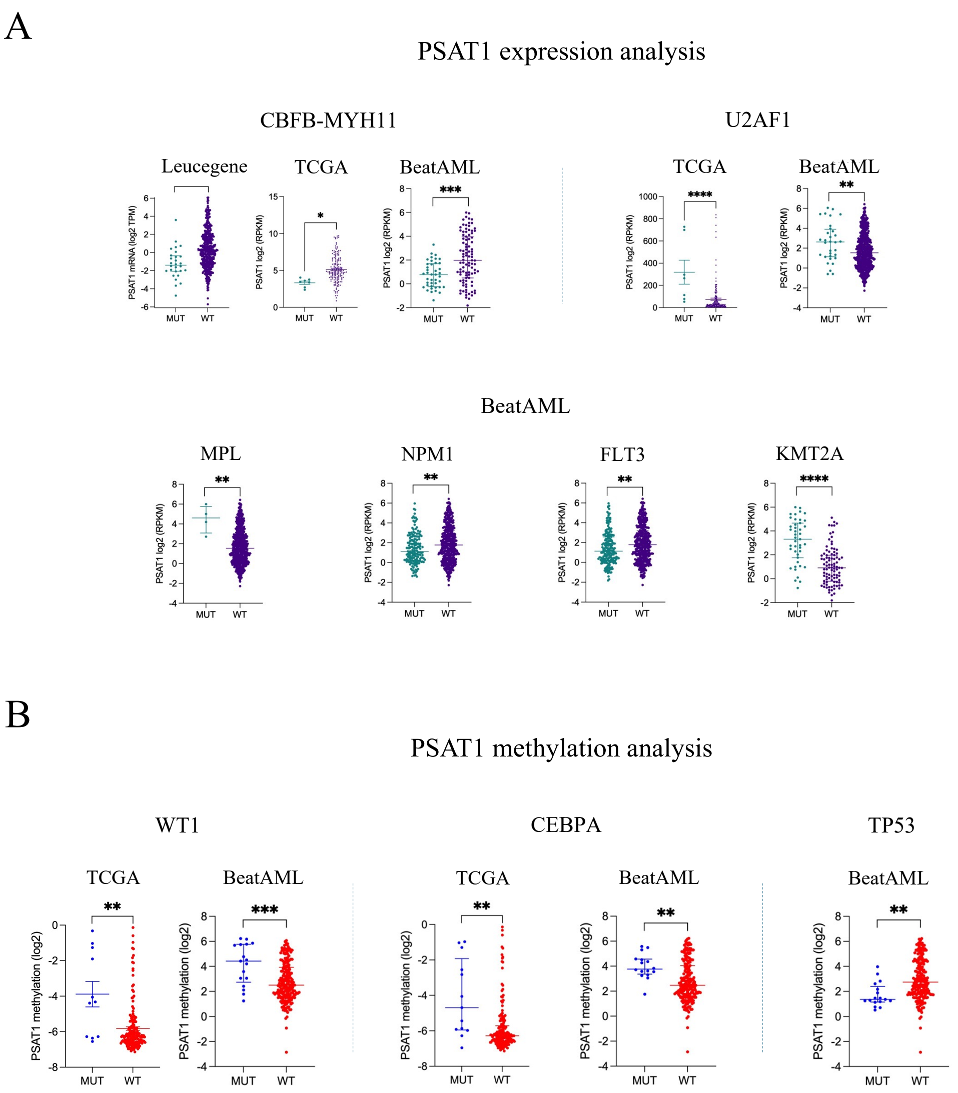

Figure S5. (A) PSAT1 expression analysis in AML patient samples harboring mutations in CBFB-MYH11, U2AF1, MPL, NPM1, FLT3, and KMT2A, using publicly available datasets from BeatAML, TCGA, and Leucegene. (B) PSAT1 promoter methylation analysis in AML patient samples with WT1, CEBPA, and TP53 mutations from the BeatAML and TCGA datasets.

Supplementary Figure 6

A

p7314

+S -S

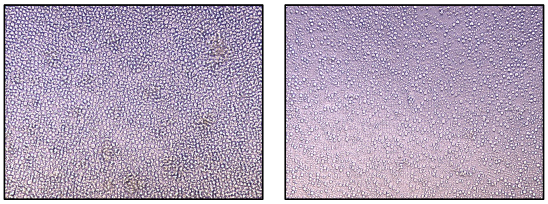

B

	Cytogenetics	Mutations
p2112	add(2)(q31),der(3)t(3;8)(q21;p21),der(8)t(2;8)(q?31;<br>p21)[20]	PTPN11 p.G60V (9.81%)<br>SF3B1 p.K700E (41,71%)
p7314	-7[16]/46,idem,+mar[4]<br>3q26(MECOM sep)*<br>-7(D7Z1,D7S486)x1*	-
p5548	i(17)(q10[13]/47,sl,+mar[7]	KIT p.D816V (10.66%)<br>TP53 p.D281N (97.54%)
p4554	normal	DNMT3A p.F752V (46.69%)<br>GATA2 p.A372T (32.02%)<br>NF1 p.S2414fs (23.54%)<br>GATA2 p.G200fs (11.66%)
p4319	del(9)(q22q34)[20]	CEBPA p.Q312_K313insQ (or p.Q312dup) (29.05%)<br>WT1 p.S444fs (35.11%)<br>EZH2 p.Y244fs (36.48%)
p6965	t(2;3)(p23;q27)[14]	SF3B1 p.K700E (31.04%)
p4229	trisomy 8 (7% nuclei)*	WT1 c.1340-2A>G (splicing variant) (6.69%)
p4676	normal	GATA2 p.A372T (32.06%)**<br>WT1 p.R441P (46.03%)**<br>WT1 p.S364fs (7.17%)**
p4359	inv2(p21q13)[20]	FLT3 p.D835E (40.18%)<br>NPM1 p.W288fs (23.89%)<br>IDH2 p.R140Q (9.12%)<br>IDH1 p.R132C (35.31%)<br>DNMT3A p.R882H (46.15%)

C

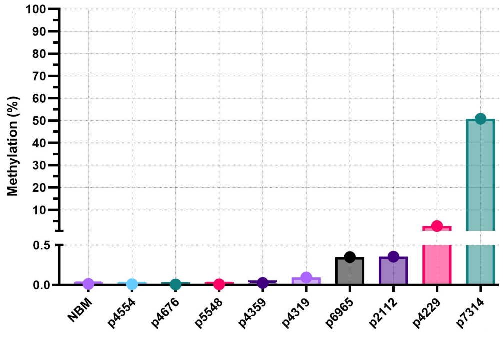

Figure S6. (A) Microscopy images showing a marked reduction in viability of the p7314 AML patient sample after 48h of incubation in +S and –S RPMI. (B) Cytogenetic (karyotype) and molecular data (NGS) from AML patient samples. The chromosomal translocation t(2,3)(p23;q27) in p6965 leads to an atypical MECOM rearrangement as described in the literature (7). *FISH findings, **NGS report obtained six months after initial sample collection. (C) Baseline DNA methylation levels (%) in AML patient samples, assessed by qPCR using the ΔΔCt method, comparing amplification from methylation-specific and unmethylation-specific primers. Each sample was analyzed in triplicate.

References

1. Luciani GM, Xie L, Dilworth D, Tierens A, Moskovitz Y, Murison A, et al. Characterization of inv(3) cell line OCI-AML-20 with stroma-dependent CD34 expression. Exp Hematol. 2019 Jan;69:27–36.

2. Kunimoto H, Fukuchi Y, Murakami K, Ikeda J, Teranaka H, Kato I, et al. Establishment of a High-risk MDS/AML Cell Line YCU-AML1 and its Xenograft Model Harboring t(3;3) and Monosomy 7. HemaSphere. 2020 Oct;4(5):e469.

3. Schindelin J, Arganda-Carreras I, Frise E, Kaynig V, Longair M, Pietzsch T, et al. Fiji: an open-source platform for biological-image analysis. Nat Methods. 2012 Jul;9(7):676–82.

4. Montrose DC, Saha S, Foronda M, McNally EM, Chen J, Zhou XK, et al. Exogenous and Endogenous Sources of Serine Contribute to Colon Cancer Metabolism, Growth, and Resistance to 5-Fluorouracil. Cancer Res. 2021 May 1;81(9):2275–88.

5. Giacopelli B, Wang M, Cleary A, Wu YZ, Schultz AR, Schmutz M, et al. DNA methylation epitypes highlight underlying developmental and disease pathways in acute myeloid leukemia. Genome Res. 2021 May;31(5):747–61.

6. Choi BH, Rawat V, Högström J, Burns PA, Conger KO, Ozgurses ME, et al. Lineage-specific silencing of PSAT1 induces serine auxotrophy and sensitivity to dietary serine starvation in luminal breast tumors. Cell Rep. 2022 Jan 18;38(3):110278.

7. Stevens-Kroef M, Poppe B, van Zelderen-Bhola S, van den Berg E, van der Blij-Philipsen M, Geurts van Kessel A, et al. Translocation t(2;3)(p15-23;q26-27) in myeloid malignancies: report of 21 new cases, clinical, cytogenetic and molecular genetic features. Leukemia. 2004 Jun;18(6):1108–14.
